# Acquisition of Subcortical Auditory Potentials With Around-the-Ear cEEGrid Technology in Normal and Hearing Impaired Listeners

**DOI:** 10.3389/fnins.2019.00730

**Published:** 2019-07-16

**Authors:** Markus Garrett, Stefan Debener, Sarah Verhulst

**Affiliations:** ^1^Department of Medical Physics and Acoustics, University of Oldenburg, Oldenburg, Germany; ^2^Cluster of Excellence “Hearing4all”, Oldenburg, Germany; ^3^Neuropsychology Laboratory, Department of Psychology, University of Oldenburg, Oldenburg, Germany; ^4^Department of Information Technology, Hearing Technology @ WAVES, Ghent University, Ghent, Belgium

**Keywords:** EEG, cEEGrid, around-the-ear EEG, auditory brainstem response, envelope following response, diagnostics, sensorineural hearing loss, auditory evoked potentials

## Abstract

Even though the principles of recording brain electrical activity remain unchanged since their discovery, their acquisition has seen major improvements. The cEEGrid, a recently developed flex-printed multi-channel sensory array, can be placed around the ear and successfully record well-known cortical electrophysiological potentials such as late auditory evoked potentials (AEPs) or the P300. Due to its fast and easy application as well as its long-lasting signal recording window, the cEEGrid technology offers great potential as a flexible and ‘wearable’ solution for the acquisition of neural correlates of hearing. Early potentials of auditory processing such as the auditory brainstem response (ABR) are already used in clinical assessment of sensorineural hearing disorders and envelope following responses (EFR) have shown promising results in the diagnosis of suprathreshold hearing deficits. This study evaluates the suitability of the cEEGrid electrode configuration to capture these AEPs. cEEGrid potentials were recorded and compared to cap-EEG potentials for young normal-hearing listeners and older listeners with high-frequency sloping audiograms to assess whether the recordings are adequately sensitive for hearing diagnostics. ABRs were elicited by presenting clicks (70 and 100-dB peSPL) and stimulation for the EFRs consisted of 120 Hz amplitude-modulated white noise carriers presented at 70-dB SPL. Data from nine bipolar cEEGrid channels and one classical cap-EEG montage (earlobes to vertex) were analysed and outcome measures were compared. Results show that the cEEGrid is able to record ABRs and EFRs with comparable shape to those recorded using a conventional cap-EEG recording montage and the same amplifier. Signal strength is lower but can still produce responses above the individual neural electrophysiological noise floor. This study shows that the application of the cEEGrid can be extended to the acquisition of early auditory evoked potentials.

## Introduction

The use of EEG as a clinical tool has a long tradition and is well established in research. Applications include (pre-surgical) diagnosis of epilepsy, the investigation of functional brainstem disorders, and degenerative illnesses (Zschocke et al., 2012). While the principles of recording the brain electrical activity remain unchanged since their discovery by Hans Berger (1924), their acquisition and the availability of EEG systems has seen major improvements. EEG acquisition devices have become smaller and mobile recording solutions are now available. Different research platforms have been developed to explore ear-centred sensors as a way of recording low-cost, motion tolerant, unobtrusive, and highly portable neurophysiological signals (Bleichner and Debener, 2017). The ear-EEG platform (Looney et al., 2012) uses EEG sensors embedded on a personalised earpiece in the ear canal and on the concha. Conductive gel (Kidmose et al., 2013) or dry electrodes (Kappel et al., 2019) are used to establish an electrode-skin connection. Other research groups have investigated the use of a soft, foldable electrode mesh that can be placed on the auricle, and mastoid (Norton et al., 2015). A third ear-centred platform, the cEEGrid, can been seen as an intermediary between in-ear and classical scalp-EEG. The cEEGrid is a flex-printed, reusable multi-channel sensor array that can be placed around the ear using adhesive and a small amount of conductive gel. Data can be recorded using regular stationary EEG amplifiers or a small mobile amplifier and commercially available smart-phones (Debener et al., 2015). These technological advances open avenues for new fields of research which require data acquisition over long periods of time or recordings outside the lab environment.

The cEEGrid technology has already demonstrated its potential for the acquisition of event related potentials (ERPs) of cortical origin, such as the N1, P1, and P300 (Pacharra et al., 2017) as well as alpha power modulation (Debener et al., 2015). Studies that directly compared cEEGrid with classical cap-EEG recordings found that cEEGrid signals had similar morphologies but with lower signal strength (Pacharra et al., 2017) and that variability in their channel impedances across participants did not measurably affect the data quality (Mirkovic et al., 2016).

Despite the great effort that has gone into validating the use of around-the-ear sensors for cortical EEG signals, very little attention has been paid to assessing its suitability for the recording of auditory evoked potentials (AEPs) with primarily subcortical origins. Given the broad clinical areas of application of these AEPs for newborn hearing screening, audiometric threshold estimation, auditory-nerve and brainstem lesion detection, recording AEPs on around-the-ear sensors can open up new avenues for mobile hearing diagnostic systems. Even though currently available mobile amplifiers do not offer sufficiently high sampling rates and amplification for the recording of these AEPs, the cEEGrid technology, and electrode configuration can already be tested in the absence of a dedicated mobile amplifier. This is a first step towards investigating the use of the cEEGrid for recording neural correlates of hearing in clinical applications. These include long-term ambulatory monitoring of AEPs or hearing-related ‘wearable’ solutions which can support the hearing-aid fitting process by providing objective neural feedback. If successful, this might kick-start the development of a suitable portable amplifier.

A popular subcortical AEP measure is the transient-evoked auditory brainstem response (ABR, Sohmer and Feinmesser, 1967; Jewett and Williston, 1971), elicited by a click or tone burst. Its characteristic five deflections (I–V) within the first 10 ms after stimulus onset stem from aggregated neural activity from ascending relay stations of the auditory pathway (Melcher and Kiang, 1996). If instead of a transient, a sustained periodic stimulus is adopted, EEG sensors pick up neural activity which phase-locks to the periodicity of the stimulus and results in a measure known as the ‘steady-state response (SSR)’. Different naming conventions have been adopted depending on the stimulus characteristics (Kraus et al., 2017). The ‘Frequency Following Response’ (FFR, e.g., Worden and Marsh, 1968; Skoe and Kraus, 2010) describes the sustained neural activity to the stimulus frequency while neural responses which follow the stimulus’ envelope have been termed ‘envelope-following response’ (EFR, Dolphin and Mountain, 1992; Bharadwaj et al., 2014). These potentials are believed to reflect summed neural activity of different interconnected subcortical nuclei (Chandrasekaran and Kraus, 2010), even though recent MEG evidence suggests that there are additional cortical contributions to the response (e.g., Coffey et al., 2016).

While (subcortical) AEPs have for many years been adopted as an objective measure to assess hearing sensitivity (Burkard et al., 2007; Picton, 2010), they only recently became a popular diagnostic measure for suprathreshold coding deficits associated with synaptopathy (Bharadwaj et al., 2014). This rise in popularity goes back to the discovery that overexposure to noise and/or ageing can lead to a loss of synapses and cochlear nerve terminals innervating the inner hair cells (IHC) (i.e., synaptopathy: Schmiedt et al., 1996; Kujawa and Liberman, 2009; Furman et al., 2013). Studies in post-mortem human temporal bones confirm that neural fibre loss might be a contributor to suprathreshold hearing deficits by showing an average loss of approximately 100 spiral ganglion cells (SGC) per year of life despite intact populations of hair cells (Makary et al., 2011) and that neural loss greatly exceeds IHC loss in most humans over the age of 60 years (Wu et al., 2018). Synaptopathy is not picked up by audiometric or ABR thresholds but is reflected in different facets of the ABR (e.g., Liberman et al., 2016; Mehraei et al., 2016; Bramhall et al., 2017; Valderrama et al., 2018) and yields reduced temporal coding fidelity as assessed by the EFR (e.g., Bharadwaj et al., 2015; Shaheen et al., 2015; Parthasarathy and Kujawa, 2018). Synaptopathy has been associated with reduced behavioural temporal processing as measured using temporal envelope sensitivity (Bharadwaj et al., 2015; Verhulst et al., 2016) and interaural time discrimination (Mehraei et al., 2016). Additionally, it is thought that these suprathreshold hearing deficits might lead to an increased difficulty to follow speech in challenging listening situations such as multi-talker scenarios (Liberman et al., 2016) as reported by many people with and without normal audiometric thresholds. The prevalence of people seeking clinical advice due to difficulties in communication in challenging listening environments, despite having normal audiometric thresholds, is estimated to be between 0.5 and 1.0% of the general population (Hind et al., 2011). This illustrates the urgency to develop objective diagnostic measures to quantify suprathreshold-deficits and synaptopathy in humans. To be of relevance for clinics, it is also important to verify that these suprathreshold EEG measures can be acquired in clinical populations with sensorineural hearing loss.

Previous research testing the feasibility of the ear-EEG platform to record ABR signals found that the responses were comparable to traditional tiptrode electrode recordings and that particularly the Wave V was clearly identifiable in the recordings. Nevertheless, the resulting wave amplitudes of the in-ear-electrodes were slightly smaller than the tiptrode potentials and the study did not present a complete in-ear measurement because external electrodes were also used (Hyvärinen et al., 2018). Regarding EFRs, a study comparing cap-EEG with ear-EEG showed that in-ear sensors can capture EFRs to diotic amplitude-modulated white noise stimuli with low modulation frequencies (40 and 80 Hz) well, even though the extracted magnitudes were 15 to 20 dB lower for within-ear recordings. This decrease in magnitude was likely due to the small distances between electrodes in the in-ear configuration. However, the signal-to-noise ratio (SNR) was comparable between recording modalities (Kidmose et al., 2013). A different ear-EEG study showed that the signal strength of ear-centred recordings was better for signals stemming from brain regions in close proximity to the sensors (Mikkelsen et al., 2015). Considering that the cEEGrid sensors are positioned in close proximity to the primary subcortical ABR and EFR generators, around-the-ear-electrodes might be able to acquire brain signals with subcortical components despite their low amplitudes. The larger inter-electrode distance of the cEEGrid compared to the ear-EEG provides the former configuration with more spatial information, which might help to identify the response features.

The purpose of this study is to evaluate the suitability of the cEEGrid electrode configuration to record early AEPs and to compare them to classical cap-EEG recordings. We use stimuli of different intensities and evaluate these measures in a reference group of young normal-hearing participants and a clinical sample of older participants with high-frequency sloping audiograms. By exploring the feasibility of the cEEGrid in recording from deep subcortical brain structures in a controlled research setting, we set out to determine its diagnostic value, and lay the foundation for future research to develop mobile cEEGrid recording solutions for audiological applications.

## Materials and Methods

### Participants

The study included 14 young normal-hearing (yNH) participants between the age of 20 and 28 (mean = 24.5 ± 2.26, 7 females) and 13 older hearing-impaired participants (oHI) with ages between 61 and 68 (mean = 65.2 ± 1.83, 8 females). One yNH participant was excluded due to strong muscle activity in the cEEGrid recordings (see section “Muscle Artifacts: Challenges and Opportunities for Around-the-Ear Sensing of Auditory EEG Measures” for discussion). Stimuli were presented monaurally on the audiometrically better ear (yNH: 9 right; oHI: 10 right) and the cEEGrid was placed on the side of the stimulated ear. All yNH participants had normal pure-tone thresholds (≤20 dB HL) assessed with a clinical audiometer (Auritec AT900, Hamburg, Germany) for frequencies between 0.125 and 8 kHz. Their average threshold at 4 kHz was 3.57 ± 3.63 dB HL. The oHI participants had high-frequency sloping audiograms with a 4 kHz-average-threshold of 37.69 ± 6.65 dB HL.

### Stimuli and Setup

Auditory brainstem response stimuli consisted of alternating polarity (condensation and rarefaction) clicks of 80-μs duration. Two different peak-equivalent sound pressure levels (peSPL) conditions: 70 and 100-dB peSPL were applied. 3000 clicks were presented at an average rate of ∼10 Hz per condition. The inter-stimulus-interval (ISI) included a short, uniformly distributed random silence jitter (mean ISI: 100 ms ± 10 ms).

The EFR stimulation consisted of two amplitude-modulated white noise stimuli with different bandwidths and a modulation frequency (*f*_m_) of 120 Hz. The broadband (BB) stimulus was band-pass filtered between 50 Hz and 16 kHz and presented at 70-dB SPL. The 4-kHz narrowband (NB) stimulus was centred around 4 kHz, one octave wide (2.8–5.7 kHz) and had an identical spectral magnitude to the band-pass filtered BB signal. Both raw noise signals were zero-phase filtered before the modulation was applied, using a Blackmann-Harris window with a filter-order of 1024 to minimise the side lobe levels. Due to the reduced bandwidth of the NB stimulus, while retaining the same spectral magnitude (calibration value) as the BB signal, the NB stimulus presentation level was slightly lower than the 70-dB SPL of the BB stimulus. A modulation depth of 95% was applied to avoid silence gaps in each modulation cycle and minimise on/offset responses. Both stimuli were ramped using a 2.5% tapered-cosine window, lasted 400 ms and were repeated 1000 times. The ISI included a uniformly distributed random silence jitter (mean ISI: 100 ms ± 10 ms).

All stimuli were generated in MATLAB at a sampling rate of 48 kHz and calibrated using an oscilloscope (for ABR only), B & K type 4157 ear simulator and sound level metre type 2610 (Brüel & Kjær, Nærum, Denmark). The digital stimuli were converted using the open-source portaudio playrec ASIO codec (Humphrey, 2008) and a Fireface UCX sound card (RME, Haimhausen, Germany) which was connected to a TDT-HB7 headphone driver (Tucker-Davis, Alachua, FL, United States); offering an impedance match to the ER-2 insert earphones (Etymotic Research, Elk Grove Village, IL, United States). The ER-2 speaker boxes were wrapped in a copper-shielding (connected to ground) and placed inside a mu-metal box to avoid magnetic artifacts in the EEG recordings. Stimuli were presented monaurally using foam tips. Triggering was achieved by presenting an additional digital channel through the playrec codec, which yielded an SPDIF output on the Fireface UCX soundcard. We used a custom-build FPGA based triggerbox which uses the clock of the Fireface UCX (max latency: 1/48000 s) to convert the SPDIF output into a 5V TLL Biosemi trigger input.

EEG recording took place in a double-walled electrically shielded measurement booth (IAC acoustics, Niederkrüchten, Germany). Participants sat comfortably in a reclining chair while watching a silent movie. Cap-EEGs were recorded using a 64-channel EEG setup with Ag/AgCl electrodes and amplifier (Biosemi, Amsterdam, Netherlands) with a sampling rate of 16384 Hz and 24-bit AD conversion. The EEG caps (Easycap) had equidistant electrode spacing. The common mode sense (CMS) active electrode was located on the fronto-central midline whereas the driven right leg (DRL) passive electrode was placed on the tip of the nose of the participant.

The c-shaped, flex-printed, ten-channel cEEGrid sensor array with Ag/AgCl electrodes was placed around the ear on the ipsilateral side of the sound stimulation using adhesive and a small amount of conductive gel. cEEGrids were connected with an in-house build connector, using a mini edge card socket (SAMTEC, Indiana, United States), proprietary printed circuit board and modified flat-type active-electrodes (Biosemi). The grids were re-used up to four times and functionality checks were administered before every recording. Data were recorded via the same amplifier than the cap-EEG by using the external touch-proof connectors to connect the cEEGrid. Only six out of ten possible cEEGrid channels were recorded due to the limited number of external connectors on the amplifier. For the purpose of this study only recordings from the vertex electrode (Cz) were used for comparison to the cEEGrid recordings. The Cz electrode configuration is known to yield good signal strength for deep neural sources (Picton, 2010), allowing for a fair comparison between electrode configurations. Electrode offsets (DC values of the common mode signal) were kept below 25 mV for all electrodes. Participants were informed about the experimental procedures according to the ethical guidelines at the University of Oldenburg. Written informed consent was obtained and participants were paid for their participation.

### Data Processing and Analysis

The raw EEG recordings were preprocessed using Python [version 2.7.10 | Anaconda 2.3.0 (64-bit)^1^] and MNE-Python (version 0.9.0) (Gramfort et al., 2013, 2014). The vertex channel was re-referenced to the offline-averaged earlobe electrodes. The cEEGrid channels were re-referenced to other cEEGrid electrodes to generate nine bipolar cEEGrid channels: E5_E1, E6_E1, E8_E1, E5_E3, E6_E3, E8_E3, E5_E4, E6_E4, and E8_E4, with the first-mentioned electrode of each pair serving as the reference (e.g., E5 was the reference electrode for the channel E5_E1). Only those bipolar channels with one electrode above and one below the horizontal midline of the cEEGrid were considered. EFR data were high-pass filtered at 70 Hz to remove low-frequent artifacts and then epoched between 0 and 400 ms after stimulus onset. ABR data were first high-pass filtered at 200 Hz and then low-pass filtered at 2000 Hz using a zero-phase filtering procedure (4^*th*^ order IIR Butterworth filter). Epoching was performed on the first 20 ms after stimulus onset. Bad epochs were identified using the joint probability criteria as implemented in EEGLAB (Brunner et al., 2013), which estimates the probability distribution of values across the data epochs per channel. Epochs with a probability of occurrence, which exceeded two standard deviations from the mean of the probability distribution were rejected from all ten channels to allow a fair comparison between cap-EEG and cEEGrid by using the exact same epochs. After the rejection procedure, there were on average 904 ± 53 epochs left for EFR computation and 2505 ± 210 for the ABR computation per participant in the yNH group. On average 916 ± 42 epochs remained per oHI participant for the EFRs and 2646 ± 183 for the ABRs.

**FIGURE 1:**
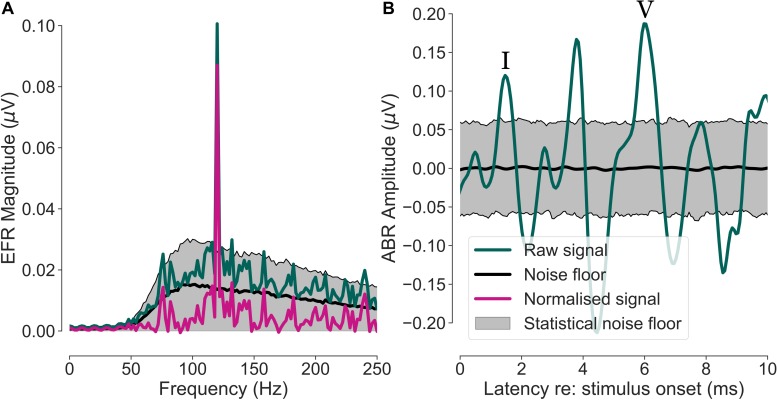
Envelope following response (EFR) and auditory brainstem response (ABR) extraction procedure for one representative yNH participant. The shown data are from the cap-EEG reference channel (averaged earlobes – vertex). **(A)** Neural response spectrum of the EFR (green) with a visible peak at the modulation frequency (*f*_m_ = 120 Hz). The purple trace shows the spectrum normalised by the noise-floor estimate (black). The grey area depicts the statistical noise floor with a significance level of α = 0.05. **(B)** Time-domain ABR signal (green) and noise-floor estimate (black) with its statistical noise floor limits (grey area, α = 0.05). Wave I and V are marked with roman numerals.

**FIGURE 2:**
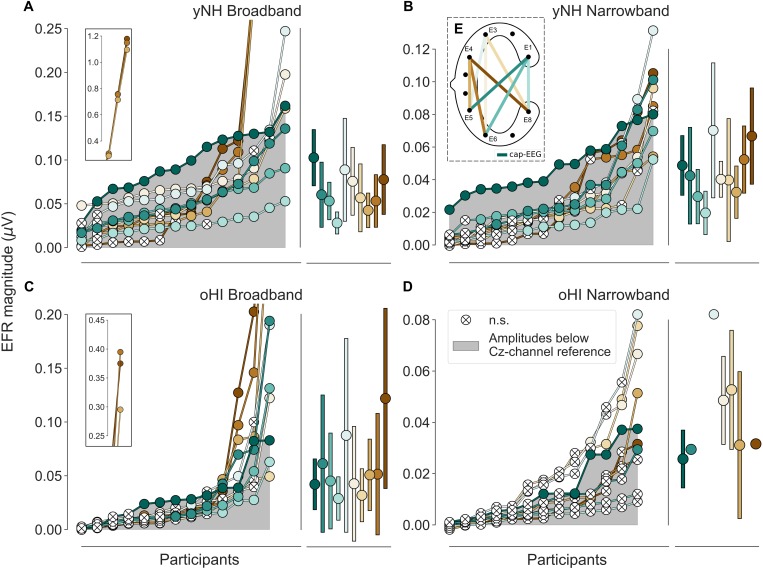
Envelope following responses ordered by magnitudes per group (yNH and oHI) and condition (BB and NB). The grey area depicts magnitudes below the cap-EEG reference channel (Cz). Crossed white points represent non-significant responses. Error bars show mean and standard deviation for all significant data points. Data points displayed on the insets in **(A,C)** were excluded from all further analyses. **(E)** Schematic of a cEEGrid, computed bipolar channels, and their colour coding. Colour code in **(E)** and legend in **(D)** apply to all panels.

A bootstrap procedure was applied to estimate the EFR and ABR signals, as well as their respective noise floors (Zhu et al., 2013). For the EFR, a magnitude spectrum estimate of the neural responses for each condition and participant was computed after averaging 700 randomly drawn epochs (with replacement). Including a fixed number of averages compensated for the different numbers of remaining epochs after noise rejection, that would otherwise influence the SNR (Luck, 2005). Identical epochs were drawn for all ten channels and this epoch-drawing and magnitude-spectrum calculation step was repeated 200 times, resulting in a distribution of magnitude spectra. The average spectrum of this approximately Gaussian-distributed measure was used as an estimate of the participant’s magnitude spectrum. The standard deviation of the 200 estimates was used as an estimator of the variability. The spectral magnitude of the noise floor was calculated using a similar approach, except that the epoch-drawing, and magnitude-spectrum step was repeated 1000 times and that the phase of half of the randomly drawn epochs was flipped (Schimmel, 1967). This method cancels out the constant time-locked signal (i.e., the EFR) in the recording and only preserves the non-stationary noise that has a characteristic shape proportional to 1/f (Voytek et al., 2015). Finally, the EFRs were normalised by subtracting the noise floor from the signal (peak-to-noise floor EFR) and the resulting magnitude peak at the modulation frequency of 120 Hz is from here on referred to as the EFR magnitude. The normalisation to the individual noise floor allows for a fair comparison of EFRs between participants and recording channels by taking individual noise-floor differences into account. Responses were considered as significantly above the noise floor if their magnitude exceeded the 1000 computed noise-floor estimates in more than 95% of all cases (statistical noise floor). Figure 1A depicts the relation between measures computed during the EFR extraction process for the cap-EEG channel of one representative yNH participant. Figure 2E shows a schematic of the electrode channels for a cEEGrid applied to the right ear. Left ear cEEGrid channels were named, respectively.

To assess the overall signal quality of the cEEGrid recordings in comparison to the cap-EEG, the SNR was calculated for all channels per condition. The SNR was computed by dividing the uncorrected bootstrapped EFR magnitudes at the modulation frequency (*f*_m_ = 120 Hz) by the corresponding bootstrapped noise-floor estimate scaled to dB: 20^*^log10 (raw signal/noise floor). To investigate the degree to which both recording modalities conveyed similar information, correlations between the cap-EEG, and each of the nine cEEGrid channel EFRs were computed for the two stimulus conditions (BB and NB), if enough data points above the statistical noise floor were available.

For the ABR, a similar time-domain bootstrapping approach was adopted (see Figure 1B). Epoch draws were repeated 200 times for the signal and 1000 times for the noise-floor estimate. The estimates were each constructed from 1000 randomly drawn (with replacement) condensation and rarefaction clicks. Half the epochs (500 per polarity) were multiplied by −1 before averaging to estimate the noise floor. ABR Wave I and V were considered for analysis and their peak-latencies and peak-to-baseline amplitudes were extracted by visual inspection for all ten channels using customised MATLAB scripts. All ABR traces per participant/channel were displayed simultaneously in a stacked format and additional information about normative latencies (Picton, 2010 – Table 8-1) and individual noise-floor levels were superimposed to guide peak extraction. For a similar approach, see the semi-automated peak picking software by Brad Buran^2^. Only data points above the statistical noise floor were considered for the analyses. All reported latencies were compensated for by the fixed recording delay of the sound delivery system (1.16 ms).

The assumptions for the performed statistical inference tests were tested using the ‘SciPy’ python package for scientific computing (Oliphant, 2007; Millman and Aivazis, 2011). The assumption of normal-distribution was tested using the Shapiro-Wilk-Test. The equal-variance assumption was tested using the Levene-Test. If assumptions were satisfied, dependent/independent *t*-tests were used to test differences between two samples. If the normal-distribution assumption was not met for two independent samples, the non-parametric Mann-Whitney *U*-Test (U) was applied. If only the equal-variance assumption was violated, Welch’s *t*-test was performed. If two dependent samples violated the normal-distribution assumption, the Wilcoxen signed-rank test (W) was applied. All correlations reported refer to the Pearson correlation coefficient (*r*) if both variables were normally distributed, otherwise Spearman’s rank correlation coefficient (ρ) was used. When multiple statistical comparisons between the cEEGrids were made, the significance level was Bonferroni adjusted for the number of tested bipolar cEEGrid channels (α = 0.05/9) to control for the family-wise error rate.

## Results

To evaluate the general applicability of the cEEGrid for recording ABRs and EFRs, we first consider the group means. Afterwards we evaluate the recorded measures on a single subject level.

### Group Level EFR

We first ensured that the reference cap-EEG recordings were of good quality and studied the expected trends between conditions and groups. The cap-EEG EFR measures were above the statistical noise floor for ∼93 and 100% of participants in the BB and NB condition, respectively. The oHI-EFRs were significant for the majority of participants (∼69%) in the BB and for nearly half of the participants (∼46%) in the NB condition (see brackets in Table 1). yNH-EFR magnitudes were significantly larger in the BB conditions [*t*_(12)_ = 10.2, *p* < 0.0001] and decreased with decreasing bandwidth of the input stimulus. The oHI group showed the same trend without significant differences [*t*_(4)_ = 2.4, *p* = 0.0745]. oHI participants showed significantly smaller EFR magnitudes for both stimulus conditions than the yNH group [BB: *U* = 8.00, *p* = 0.0004, *n*_1_ = 13, *n*_2_ = 9; NB: *t*_(18)_ = 2.8, *p* = 0.0118]. These trends follow well-documented observations in the EFR literature (e.g., Purcell et al., 2004; Garrett and Verhulst, 2019).

**TABLE 1:** Average EFR signal-to-noise ratio (SNR) in dB for the cap-EEG (Cz) and cEEGrid channels, conditions (BB and NB), and participant groups (yNH and oHI) including all data points.

	**Cz**	**E5_E1**	**E6_E1**	**E8_E1**	**E5_E3**	**E6_E3**	**E8_E3**	**E5_E4**	**E6_E4**	**E8_E4**
yNH BB	19.5 (93)	12.0 (**93**)	**12.6** (**93**)	10.3 (86)	9.8 (79)	10.2 (**93**)	8.2 (79)	8.2 (50)	9.7 (64)	7.3 (36)
yNH NB	14.7 (100)	**8.2** (71)	8.1 (**86**)	6.7 (64)	4.7 (36)	4.4 (29)	3.7 (27)	5.6 (43)	6.5 (43)	5.6 (36)
oHI BB	8.7 (69)	**6.5** (**54**)	6.0 (46)	5.4 (38)	5.0 (23)	4.5 (31)	3.0 (15)	5.6 (38)	6.0 (46)	5.5 (23)
oHI NB	4.9 (46)	3.0 (8)	2.2 (0)	1.7 (0)	**3.6** (8)	3.3 (**23**)	3.3 (**23**)	3.1 (15)	2.4 (0)	2.2 (8)

*The values in brackets show the number of significant responses in percent. Bold values indicate the channel with the largest response for each row, respectively.*

For each channel the extracted EFR magnitudes of all participants per group and condition were ranked in ascending order to compare the performance of the cEEGrid vs. the cap-EEG as depicted in Figure 2 (note the different *y*-axis scaling). There were three yNH and one oHI participant who showed unusually large magnitudes for three particular bipolar channels including electrode E4 in the BB condition (see insets of Figures 2A,C). These data points were removed for the remaining analyses as they may have been compromised by muscle artifacts (see section “Muscle Artifacts: Challenges and Opportunities for Around-the-Ear Sensing of Auditory EEG Measures” for discussion). cEEGrid channels showed smaller magnitudes for the narrower stimulus bandwidth condition, which was also observed in the cap-EEG recordings. All data points with a positive SNR (including those below the statistical noise floor) were used to test the differences between stimulus conditions and groups statistically. For the yNH group, cEEGrid channels E5_E1, E6_E1, E8_E1, E5_E3, E6_E3, and E8_E3 (0.0010 ≤ *p* ≤ 0.0035) decreased significantly between conditions. Only channel E6_E1 (W = 5, *p* = 0.0046, *n* = 13) showed a significant difference for the oHI group. Between groups, the cEEGrid channels E6_E1, E5_E3, E6_E3, and E8_E3 (0.0001 ≤ *p* ≤ 0.0026) showed significant differences in the BB conditions and channels E5_E1, E6_E1, E8_E1, and E6_E4 (0.0001 < *p* ≤ 0.0030) in the NB condition. The majority of cEEGrid-EFRs had smaller magnitudes than the cap-EEG (grey area) in both participant groups. Nevertheless, there were few participants with cEEGrid magnitudes above the cap-EEG reference magnitudes. Particularly for the NB condition compared to the BB condition, a growing number of cEEGrid channel magnitudes were below the statistical noise floor, showing a generally reduced sensitivity of the cEEGrid to recording EFR measures compared to classical cap-EEG.

In three out of four conditions, the diagonal-forehead directed channel E5_E3 showed overall the largest magnitude for all data points above the statistical noise floor (see error bars in Figure 2 and brackets in Table 1 for the percentage of included data points). For the oHI-BB condition, only channel E8_E4 presented a higher average magnitude. Possible muscle-artifact related reasons for these elevated magnitudes are discussed in section “Muscle Artifacts: Challenges and Opportunities for Around-the-Ear Sensing of Auditory EEG Measures.” The cEEGrid channels with the most significant responses showed a similar trend across conditions (see brackets in Table 1). In both groups, diagonal channels (directed towards the forehead: E5_E1, E6_E1) and a vertical channel with the highest inter-electrode distance (directed towards the top of the head; E6_E3) showed the most measurable responses. The very few significant responses in the oHI-NB condition (2–3 participants) also favoured a backward-tilted channel (E8_E3). For the yNH-BB group, we found significant correlations for channels E5_E1 (*r* = 0.59, *p* = 0.034, *n* = 13), E5_E3 (ρ = 0.66, *p* = 0.026, *n* = 11), and E6_E4 (*r* = 0.82, *p* = 0.013, *n* = 8) but none for the yNH-NB condition. For the oHI group, a correlation analysis was not performed due to the low number of data points above the statistical noise floor. If the correlation was based on all, not just the significant data points for the oHI-BB condition, we see a significant correlation for channel E8_E3 (ρ = 0.67; *p* = 0.012; *n* = 13) while channel E5_E3 (ρ = 0.54; *p* = 0.055; *n* = 13) showed a similar but not significant trend. In the oHI-NB condition we did not find significant correlations for any channel when including all data points. When comparing the SNRs between recording modalities, the cap-EEG showed the highest average SNRs for all conditions. For the cEEGrids a diagonal-forehead directed channel (E5_E1, E6_E1, and E5_E3) achieved the highest SNR in all conditions (see bold numbers in Table 1). Note that the excluded E4 channels (Figure 2) were not included in the SNR computation.

### Group Level ABR

**FIGURE 3:**
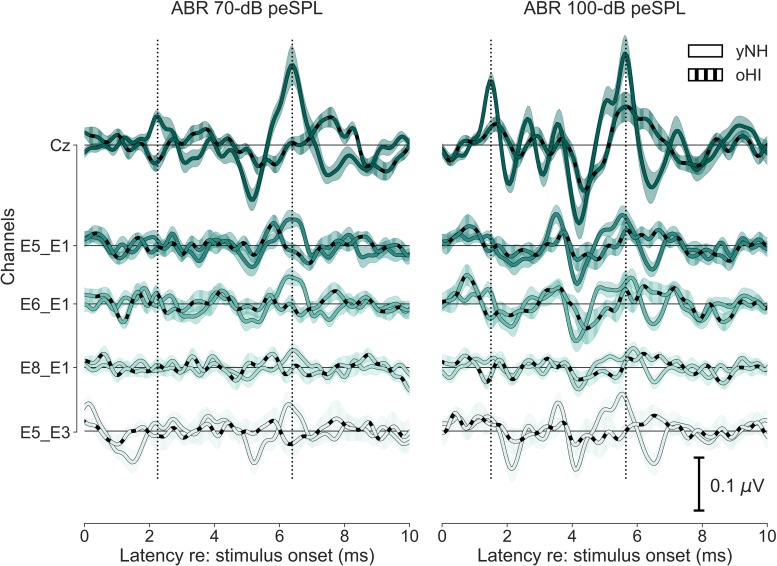
Grand-average time-domain waveforms of five ABR channels (Cz, E5_E1, E6_E1, E8_E1, and E5_E3) for both levels (70 and 100-dB peSPL) and participant groups (yNH and oHI). Error margins depict the standard error of the mean. Vertical dotted lines mark the latencies of Wave I and V in the cap-EEG channel (Cz) for the yNH participant group.

The Wave-I and V characteristics were most pronounced in the 100-dB peSPL cap-EEG condition (Figure 3) and Wave V was also visible in the cEEGrid channels for the yNH group. Wave I, where present, was much less prominent compared to the cap-EEG Cz-channel recording. For the oHI group, the waves are hard to distinguish from the noise for the displayed averaged traces and channels. For the 70-dB peSPL cap-EEG condition, the Wave I was above the statistical noise floor in ∼86% of yNH participants and in only ∼15% of the oHI participants. The more pronounced Wave V was identified in 100% of yNH and ∼62% of oHI participants, respectively. Identification rates increased with increasing SPL. In ∼93% percent of the yNH and ∼46% of the oHI participants, Wave I had amplitudes which significantly exceeded the statistical noise floor (see Table 2).

**TABLE 2:** Number of significant ABR responses in percent that were extracted from the data for each channel (cap-EEG and cEEGrid), condition (70 and 100-dB peSPL), wave (I and V), and participant group (yNH and oHI).

	**Cz**	**E5_E1**	**E6_E1**	**E8_E1**	**E5_E3**	**E6_E3**	**E8_E3**	**E5_E4**	**E6_E4**	**E8_E4**
yNH I 70	86	7	**43**	14	7	14	7	0	14	0
yNH V 70	100	**57**	**57**	43	**57**	50	36	50	43	36
yNH I 100	93	14	29	**36**	14	21	**36**	7	14	21
yNH V 100	100	**79**	**79**	43	57	64	43	50	50	43
oHI I 70	15	0	8	0	0	0	0	**15**	**15**	0
oHI V 70	62	15	**23**	15	**23**	**23**	**23**	8	0	0
oHI I 100	46	8	8	**31**	15	0	15	23	8	8
oHI V 100	100	23	**38**	31	23	31	31	15	31	15

*Bold values indicate the highest cEEGrid-percentage for each row.*

**FIGURE 4:**
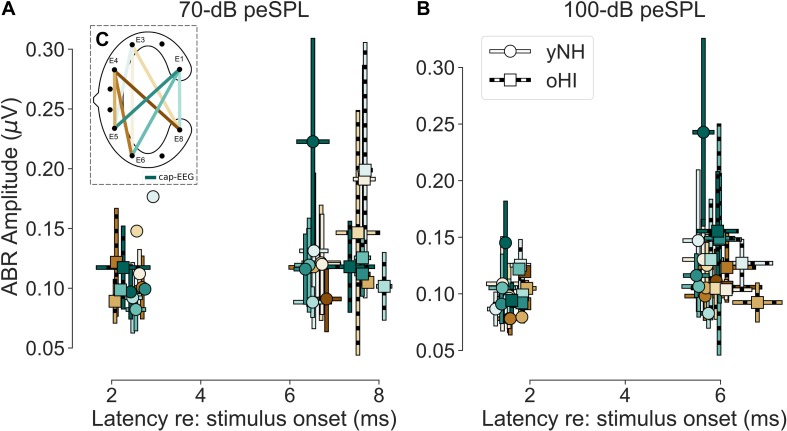
**(A**,**B)** Mean amplitudes and peak-latencies of the extracted significant ABR Wave I and V for both levels (70 and 100-dB peSPL) and participant groups (yNH and oHI). Error bars show the standard deviation. Colour code in **(C)** and legend in **(B)** apply to all panels.

For the ABR Wave V, all participants showed measurable and significant wave-peaks (Figure 4). For the cap-EEG reference channel within a group, the only significant increase in amplitude between SPLs was found for the yNH Wave I [*t*_(10)_ = −4.0, *p* = 0.0026]. Between groups, the Wave I [100 dB: *t*_(17)_ = 3.2, *p* = 0.0050] as well as Wave V [70 dB: *U* = 10.0, *p* < 0.0009, *n*_1_ = 14, *n*_2_ = 8; 100 dB: *U* = 23.0, *p* < 0.0005, *n*_1_ = 14, *n*_2_ = 13] showed significantly larger responses for the yNH group. For the cEEGrid-ABR, it can be noted that the percentage of participants who showed detectable significant responses was always below that of the cap-EEG data. Additionally, the percentage was always smaller for the oHI group. In many channels and especially for the lower SPL condition and oHI group, the cEEGrid was not sensitive enough to record significant responses.

There were clear trends in the data regarding the channels which showed the largest number of measurable responses (see Table 2). For all Wave-V conditions, independent of group and SPL, the diagonal-forehead directed channel E6_E1 was amongst the best channels. For the Wave I, the trend was not as clear as for the Wave V, but in three out of four conditions (including both 100-dB SPL conditions) a vertical channel with medium distance between electrodes (E8_E1, E5_E4) was amongst the best configurations. Especially for the 100-dB peSPL condition, we observed wave-specific orientations that maximised the likelihood to pick up a signal. The diagonal-forehead directed channel E6_E1 was particularly suitable to capture the Wave V, while the vertical channel E8_E1 had the highest rate of significant responses for Wave I.

There was no single channel that maximised the ABR across waves, which is not surprising given that the waves stem from different generators along the auditory pathway (Melcher and Kiang, 1996). There were no clear group effects, but there were some differences between waves. For Wave V, the best channel was always a diagonal-forehead directed channel (E5_E3 or E5_E1) on a group level, whereas for the Wave I, it could also be a vertical (E6_E3), or slightly backward-tilted channel (E6_E4). There was a discrepancy between the most reliable channels and the amplitude-maximising channels, but with similar trends regarding their orientation. A correlation analysis with the yNH cap-EEG showed only one significant relation with the Wave V at 70-dB peSPL (channel E6_E3; *r* = 0.84, *p* = 0.0166, *n* = 7) and correlations were not performed for the oHI group due to the low number of significant data points. Based on the few available data for most channels, cEEGrid amplitudes were on average smaller than the cap-EEG data. However, for some channels and conditions, the cEEGrid showed on average larger amplitudes compared to the cap-EEG, as can be seen in Figure 4 and particularly for the oHI participants.

The average cap-EEG latencies generally showed significantly longer ABR Wave-I and V latencies in the oHI group compared to the yNH group in agreement with other observations (e.g., Burkard and Sims, 2002). For the 70-dB peSPL condition, Wave-I latencies were not considered for statistical comparisons due to the few available data points. For the 100-dB peSPL condition the oHI showed significantly longer Wave-I latencies [*t*_(17)_ = −2.20, *p* = 0.0420]. The same was true for Wave V at both peSPLs [70 dB: *t*_(20)_ = −4.25, *p* = 0.0004; 100 dB: *t*_(17.7)_ = −2.22, *p* = 0.0400]. Overall, the average cEEGrid channel latencies showed the same behaviour between groups as the cap-EEG (see Figure 4) but were not compared on a statistical level. Within a group, cap-EEG and cEEGrid channels showed good consistency in average latencies: for the cap-EEG data Wave I [yNH: W = 0.00, *p* = 0.0033, *n* = 11] as well as Wave V [yNH: *t*_(13)_ = 15.45, *p* < 0.0001; oHI: *t*_(7)_ = 10.25, *p* < 0.0001] showed significantly reduced latencies with increasing SPL corroborating multiple cap-EEG observations (e.g., Gorga et al., 1985; Neely et al., 2003; Lewis et al., 2015). The oHI Wave I in the 70-dB peSPL condition was not considered for statistical comparison.

### Noise-Floor Levels

**FIGURE 5:**
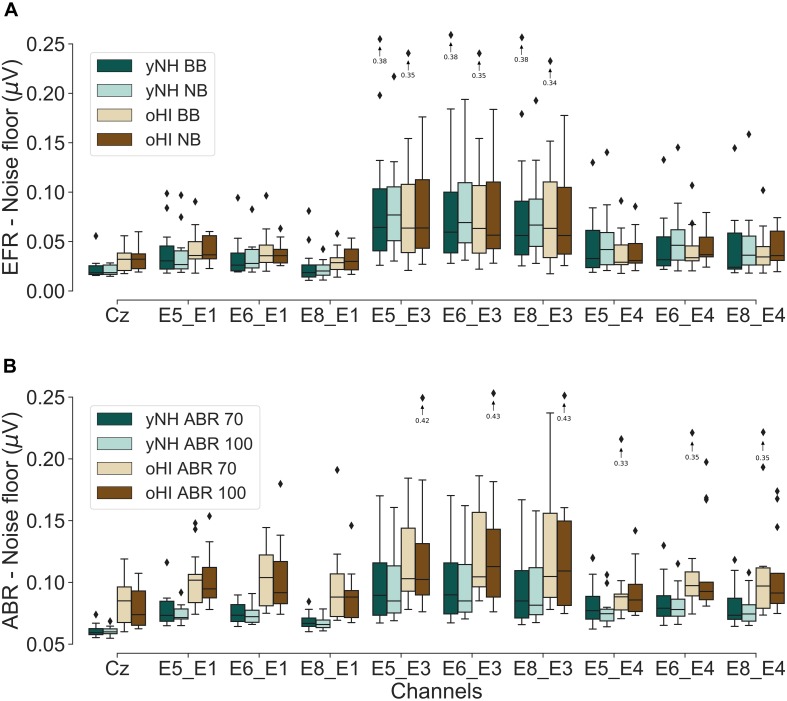
Boxplots of the estimated statistical noise-floor levels (α = 0.05) for the EFR **(A)** and ABR conditions **(B)** for both participant groups (yNH and oHI). Arrows/diamonds with associated values indicate outliers outside the displayed voltage range.

As signal detection of the EFR and ABR does not only depend on the individual signal strength but also on the level of recording noise, we investigated the noise-floor levels for all channels, metrics, and participant groups. Figure 5 depicts the statistical noise-floor levels for all data points which mark the detection threshold on a significance level of α = 0.05. As the estimated ABR noise floor could be considered constant across the whole epoch length, the mean statistical noise-floor level of the first 10 ms after stimulus onset is shown.

The EFR cap-EEG data showed that oHI participants had significantly higher noise floors in both conditions compared to the yNH group [BB: *U* = 37, *p* = 0.0047, *n*_1_ = 14, *n*_2_ = 13; NB: *U* = 29, *p* = 0.0014, *n*_1_ = 14, *n*_2_ = 13]. This was not the case for any of the cEEGrid channels. The cap-ABRs also showed higher noise floors for the oHI group [70 dB: *U* = 13, *p* = 0.0001, *n*_1_ = 14, *n*_2_ = 13; 100 dB: *t*_(13.27)_ = −4.52, *p* = 0.0006]. For the ABRs, this was also the case for channels E5_E1, E6_E1, E8_E1, and E8_E4 (0.0006 ≤ *p* ≤ 0.0031) for 70-dB peSPL and channels E5_E1, E6_E1, E8_E1, E5_E4, E6_E4, and E8_E4 (0.0002 ≤ *p* ≤ 0.0054) for the 100-dB peSPL condition.

Exemplarily for all conditions, we compared the cEEGrid noise-floor levels of the BB-EFR and 100-dB peSPL ABR condition in both groups against the cap-EEG channel. All but one cEEGrid channel (E8_E1) showed significantly higher statistical noise floors in the yNH-BB condition compared to the cap-EEG (0.0010 ≤ *p* ≤ 0.0033). For the oHI-BB condition, this was only the case for the three channels including electrode E3 (0.0015 ≤ *p* ≤ 0.0019). For the yNH-ABR data, all channels had higher noise floors than the cap-EEG data (0.0001 < *p* ≤ 0.0010). For the oHI group this was the case for all but the channels E8_E1 and E5_E4 (0.0002 ≤ *p* ≤ 0.0030).

### Individual EFR and ABR Profiles

**FIGURE 6:**
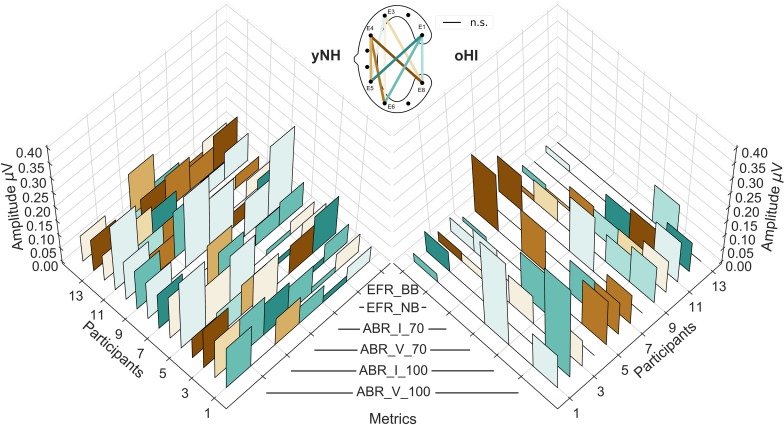
Individual cEEGrid profiles of the six investigated auditory evoked potentials for both participant groups (yNH, left; oHI, right). The EFRs for the broadband (EFR_BB) and narrowband condition (EFR_NB), the ABR amplitudes for both levels (70 and 100-dB peSPL) for Wave I (ABR_I_70, ABR_I_100), and Wave V (ABR_V_70, ABR_V_100). The height of the individual bars represent the amplitude/magnitude while the colours code the cEEGrid channel with the maximum amplitude per metric. Black lines at the 0 μV level represent non-significant data points. The corresponding cEEGrid channels represented by the colours are visualised in the schematic (top-middle).

To evaluate the EFR and ABR data on a single subject level, individual profiles consisting of the two EFR metrics (BB and NB) and the four ABR metrics (Wave-I and V amplitudes for both peSPLs) are depicted in Figure 6. Regardless of group and measure, the majority of responses showed their largest amplitudes in the diagonal-forehead directed channels (E6_E1, E5_E1, and E5_E3) and the vertical channels E8_E1, E5_E4, and E6_E3. Within each participant, the channels of maximal magnitude/amplitude varied between measures (EFR and ABR) but also between stimulus intensity levels within a measure. There were no clear patterns observed between participant profiles.

## Discussion

We investigated the feasibility of adopting a flat c-shaped around-the-ear electrode array for the recording of ABRs and EFRs in a young normal-hearing group and an elderly participant group with high-frequency sloping audiograms. We analysed nine bipolar cEEGrid channels with different orientations and compared the results to a classical cap-EEG montage. Our results showed that the cEEGrid is able to record steady-state responses, and to a lesser degree, transient responses from the brainstem in most yNH and a few oHI participants for the considered stimulus conditions and SPLs. Responses showed similar morphologies and response patterns to those observed for the cap-EEG when responses were above the statistical noise-floor level.

### Effects of Stimulus Intensity and Sensorineural Hearing Loss

The data recorded from the cEEGrid channels generally showed lower SNRs than the cap-EEG for the AEPs recorded here, which corroborates findings in previous studies for cortical potentials (Mirkovic et al., 2016; Pacharra et al., 2017). Consequently, less salient stimuli conditions with a lower bandwidth and/or lower SPL did not always exceed the individual statistical noise floor. As we only used moderate stimulus intensities (EFR: ∼ 70 dB; ABR: 70, 100 dB peSPL), detection rates are likely to increase when using higher SPLs. The reduced sensitivity of the cEEGrid is particularly important when considering people with sensorineural hearing loss. The loss of outer hair cells as evident from elevated audiometric thresholds and the possible presence of neural fibre loss decrease the input to the auditory brainstem regions and renders many responses undetectable in the cEEGrid or even cap-EEG electrode configurations. The number of non-significant responses in the considered oHI group was highest for the EFR-NB and the ABR 70-dB peSPL condition (see Figure 6). These results are likely a combination of the reduced sensitivity of the cEEGrid and the different pathologies at play.

### Noise-Floor Level Differences

The estimated noise-floor levels showed that the cEEGrid channels generally present higher statistical noise floors compared to the cap-EEG and that these levels can vary across the bipolar channels. Generally, the noise-floor levels were similar across bipolar channels but the data showed that those bipolar channels including electrode E3 presented increased variability and elevated noise-floor levels. The electrode is positioned in the small gap between the auricle and the hair line close to the anterior and superior auricular muscles and might make this channel more prone to external noise. Nevertheless, these channels (E5_E3, E6_E3, and E8_E3) showed very comparable SNRs to the other cEEGrid channels which means that the data quality was not compromised by the higher noise levels, in line with the findings by Mirkovic et al. (2016).

The noise floors for the cap-EEG data were also higher for the oHI than the yNH group, replicating the findings of other studies (e.g., Purcell et al., 2004; Picton et al., 2005; Voytek et al., 2015). For the EFR, elevated noise floors for the oHI group were only found for channel E8_E1 in the BB condition. For the transient ABR on the other hand, most bipolar channels showed increased noise levels in the oHI group. The frequency-domain EFR noise-floor estimates only depended on the noise in a very narrow frequency region around the modulation frequency whereas the ABR time-domain noise-floor estimate comprised all frequency contribution in the passband. The more dominant differences in the ABR noise floors between groups were therefore expected. The higher statistical noise floors for the oHI group, in addition to their lower signal amplitudes, help to explain the lower detection rates observed for the hearing-impaired participants.

### The Role of the Orientation of Bipolar cEEGrid Channels

In line with previous studies, we observed that the orientation of the bipolar channels and the distance between them plays a role for the signal strength and reliability of signal detection (Bleichner et al., 2016; Denk et al., 2018). Responses were very similar for channels with similar orientations for the AEP measures.

For the EFR, diagonal-forehead directed or vertical channels were most suitable and these findings are in line with mirrored dipole models of the FFR response which predict the primary generators to be located in the inferior colliculus (IC) with a voltage gradient running parallel to the brainstem (Bidelman, 2015). The cEEGrid channels are much closer to peripheral generators such as the cochlear nucleus as compared to the earlobe-vertex configuration which is positioned between peripheral and more central generators such as the IC (Coffey et al., 2016). As the amplitude of an electrical dipole decreases proportionally to the inverse of the cube of the distance to the source, we may expect that cEEGrid channels capture more peripheral sources. This might explain the absence of correlations between most bipolar cEEGrid channels and the cap-EEG. As these more peripheral sources are assumed to have a better phase-locking ability for higher signal frequencies, we should have seen increased energy at the harmonics of the fundamental frequency in the neural response spectrum compared to the cap-EEG (Bidelman, 2015). This was not the case in our data, which might also be due to the close proximity of electrode pairs in the cEEGrid channels resulting in poorer SNRs (Mikkelsen et al., 2015; Denk et al., 2018).

For the ABRs, we found that the same channels which were most sensitive to the EFR were also suitable to pick up the Wave I and V. As the different waves are known to have different generators, namely the auditory nerve (Wave I) and the IC (Wave V) (Møller and Jannetta, 1985), we expected to see different cEEGrid channel orientations to be favoured for the different waves. We only found these trends when evaluating the overall rate of significant responses per channel in the high peSPL condition. The Wave I was most reliably captured by the vertical channel E8_E1 whose electrodes presumably have the smallest Euclidean distance to the auditory nerve, while showing the smallest inter-electrode distance, and lowest noise-floor estimates of all cEEGrid channels (see Figure 5). The Wave V on the other hand favoured diagonal-forehead directed channels, similarly to the EFR which is in accordance with its primary generator stemming from the IC.

#### Variability in Optimal Channel Configuration

Even though the data showed similar trends across participants regarding the best electrode configuration, the individual profiles presented a large degree of variability between maximal response channels per participant and even within-subject stimulus conditions. This suggests that individual channels are susceptible to minor changes in the recording conditions (e.g., skin conductance) or that the relative contributions of the signal generators change for the different stimulus conditions within a metric and underlie individual differences in the participants anatomy, which influences the potential field of the metrics (Looney et al., 2012). Even though great care was taken to place the cEEGrid array in the same location and orientation for each participant, individual features such as head shape, size of the ear, and hair growth make it likely that the orientation between cEEGrid and brain structures was not always identical. Additionally, as the ABR and EFR are neural correlates of peripheral hearing, we expected to see strong individual differences in response amplitudes/magnitudes especially among the oHI participants, which is reflected in the increased variance in this group. This variability probably contributes to some part to the discrepancy observed between channels that show the highest number of significant responses, and those that maximise the amplitude.

As neural sources project differently to different locations on the head, the cEEGrid might be more suitable for some than other neural measures (Bleichner and Debener, 2017). Here we found that the cEEGrids picked up more EFRs above the statistical noise floor compared to the ABRs, suggesting that the location, and orientation of the cEEGrid is more suited for recording steady-state responses compared to transient responses. This is in line with findings in the ear-EEG literature (Kidmose et al., 2012; Mikkelsen et al., 2015).

### Muscle Artifacts: Challenges and Opportunities for Around-the-Ear Sensing of Auditory EEG Measures

Due to the positioning of the cEEGrid electrodes with respect to the anatomy of the human head, they are not only sensitive to brain activity, but also to muscle activity. The post-auricular muscle (PAM), located just behind the ear, plays a particularly interesting role for sound-evoked responses. If exposed to brief acoustic stimuli, like those adopted here for ABR stimulation, the PAM elicits the so called post-auricular muscle response (PAMR, O’Beirne and Patuzzi, 1999). It consists of a bipolar compound action potential following the ABR Wave V at around 12.5–15 ms, which has much larger amplitudes than the ABR. In our recordings, we were able to capture this potential in a few subjects but only for the three bipolar channels including electrode E4 (E5_E4, E6_E4, and E8_E4) which was positioned in close proximity to the post-auricular muscle. All other channels were largely unaffected by the PAMR. The largest PAMRs were elicited by the ABR 100-dB peSPL condition. While the PAMR elicited much larger amplitudes than the ABR (∼0.5 μV), the ABRs could still be extracted from the waveform. As it is known that the cochlea is the receptor organ that drives the PAMR, it has the potential to be used as a diagnostic measure e.g., in neonatal deafness screenings for which a cEEGrid channel can be placed on the PAM.

#### Non-neural Contributions to the cEEGrid-EFR

Even though the majority of participants showed EFR channel preferences and magnitudes within a physiologically plausible orientation and size, there were some participants that showed very large EFRs in backward-tilted and vertical cEEGrid channels, including the electrode E4 (see insets in Figure 2). These were much higher than magnitudes reported in the literature for cap-EEG (e.g., Purcell et al., 2004; Bharadwaj et al., 2015; Dimitrijevic et al., 2016; Verhulst et al., 2018) and therefore unlikely to be of neural origin. For these four participants (3 yNH, 1 oHI), the channels in question were excluded from the analyses. Due to the fact that only selected cEEGrid channels (not the cap-EEG) and a few participants were affected, we can exclude the possibility that these results are due to electrical leakage coming from the sound transducer. The fact that these large responses only appeared in the loudest EFR condition (BB) at 70-dB SPL and that a visible onset responses in the EFR time signal was present in all other channels including the cap-EEG further supports that this was a physiological response, and not an electrical artifact. One participant that was excluded from the study showed large EFRs in the BB condition as well as very strong PAMRs. All other four subjects showed only large BB-EFRs but normal ABRs. Nevertheless, given that the same three channels as for the PAMs were affected in the BB-EFRs, it seems likely that the PAM might contaminate the neural response. If this also affected the other participants to a certain degree, it might explain why some participants showed largest responses in the three E4-channels (E5_E4, E6_E4, and E8_E4) as seen in Figure 6. As the observed effect is only present for high intensity sounds (≥70 dB SPL), it might also be possible that the middle-ear-muscle reflex (MEMR) contributes to the EFRs with largest magnitudes. The MEMR has a SPL threshold between 70 and 100 dB SPL, which falls within the SPL range used for the BB-EFRs. Given that MEMR thresholds are not, or only slightly, elevated for people with noise-induced hearing loss (Niemeyer, 1971), it could explain why one oHI participant also showed unusually high EFRs.

### Around-the-Ear vs. In-Ear Recordings of AEPs

The reported results present evidence that around-the-ear sensors are suitable to record the subcortical brain activity reflected in the considered AEPs above the statistical noise floor in most yNH and some oHI participants for the same SPLs. Nevertheless and especially for ABRs, tiptrode electrodes placed within the ear canal are often used to improve the recording quality especially for Wave I (e.g., Liberman et al., 2016; Bramhall et al., 2017; Valderrama et al., 2018). Therefore, electrode locations within the ear might be more suitable for the recording of ABRs. First attempts to record ABRs with the ear-EEG platform showed a visible Wave I and prominent Wave V in NH participants (Hyvärinen et al., 2018) but these data were referenced to the fronto-central channel (Fz) or the contralateral ear, similar to classical montages. Further research is necessary to show that these results can be replicated when placing all recording channels within the ear canal or concha. The small inter-electrode distance between the ear-EEG might constitute a problem in this context by reducing the amplitude of the small wave peaks.

Ear-EEG literature generally reports smaller magnitudes but also lower noise floors for the in-ear electrodes compared to cap-EEG when examining low-modulation-frequency steady-state responses which yield comparable SNRs between ear-EEG and cap-EEG (Kidmose et al., 2012, 2013; Bech Christensen et al., 2018). Using the cEEGrid, we did not confirm these trends. The noise floor was on average higher than for the cap-EEG yielding smaller SNRs compared to the cap-reference. The higher noise floors might in part be due to the close proximity of the cEEGrid electrodes to prominent muscles such as the jaw or the auricular muscles. This might be a real advantage for the ear-EEG in recording deep neural sources as compared to the cEEGrid configuration.

Our data showed that the optimal channel configuration which achieved the highest signal quality is highly variable between participants and conditions. This has also been reported for ear-EEG (e.g., Kidmose et al., 2012; Christensen et al., 2018). The cEEGrid might therefore have an advantage for the detection of ABRs and EFRs by providing more spatial information (Bleichner and Debener, 2017) and therefore a higher likelihood to capture the underlying sources. So far, the ear-EEG platform has only been used to record EFRs with low modulation frequencies (e.g., Kidmose et al., 2013; Mikkelsen et al., 2015; Kappel et al., 2019) but has done so very successfully. It remains to be tested if the ear-EEG can record EFRs to higher modulation-frequency stimuli with comparable SNRs. The ear-EEG has also been used in participants with sensorineural hearing loss for objective threshold estimation using steady-state responses (Bech Christensen et al., 2018), a study in which increased difficulty in obtaining steady-state responses from HI compared to NH participants was also reported.

### Considerations for Future Applications

Our recordings show that the cEEGrid channels recorded measurable EFRs above the neural electrophysiological noise floor in the BB condition in 93% of all yNH and 54% of all oHI participants in the optimal channel. This is comparable to the 93% and 69% that were achieved with the cap-EEG. For the ABRs in the 100-dB peSPL conditions, the results were not as promising. Only 36% for Wave I (cap-EEG: 93%) and 79% for Wave V (cap-EEG: 100%) of yNH participant showed measurable responses. In the oHI group these numbers reduced to 31% (cap-EEG: 46%) and 38% (cap-EEG: 100%) for Wave I and V, respectively. The ear-centred sensors therefore seem to be better suited for the recording of sustained responses such as the EFR which are based on frequency-domain analyses as another study also concluded (Mikkelsen et al., 2015). For potential applications of the cEEGrid, our study suggests to use sufficiently high SPLs, especially in clinical populations with sensorineural hearing loss, to increase the probability to detect a response. Due to the reduced sensitivity, especially to ABRs, the cEEGrid seems unsuitable for several diagnostic procedures such as objective hearing threshold detection using the ABR. However, thresholds might still be extracted using a steady-state signal approach as shown in ear-EEG studies (Bech Christensen et al., 2018; Christensen et al., 2018). Another critical point is the interpretability of the cEEGrid results in relation to classically used montages. As the correlation between the cap-EEG and cEEGrid-EFR magnitudes were only significant in very few channels and only for the yNH group, the majority of cEEGrid channels might record complementary, but not identical, information. This is possibly due to a different relative sensitivity to the EFR/ABR source generators compared to the cap-EEG montage. On the other hand, the ABR latencies were strongly correlated with the cap-EEG in the majority of channels for the yNH Wave V and also for some channels for the oHI group. This means that at least for midbrain generators, the cEEGrid faithfully represents the response latency. Where data were available, the cEEGrid channels followed the same trends as the cap-EEG (see Figures 2, 4) and seemed sensitive enough to capture tested stimulus differences (bandwidth and SPL changes) at least for the yNH group. Additionally, for very few participants, some cEEGrid channels showed similar or higher amplitudes compared to the classical cap-EEG montage, suggesting that under certain conditions it is possible to achieve comparable signal quality for auditory brainstem measures using ear centred sensors.

## Summary and Conclusion

We demonstrated that the cEEGrid is able to record ABRs as well as EFRs with amplitudes/magnitudes above the statistical noise floor in a controlled lab setting, providing the proof-of-concept for future developments in amplifier technologies and mobile EEG solutions. The amount of detectable responses strongly depended on the salience of the stimulus, considered metric and the participant group, with oHI participants showing smaller amplitudes and less detectable responses. On a group level, the cEEGrid reproduced the trends seen between the stimulus conditions and participant groups in the cap-EEG reference recordings. The overall best channels among participants showing the largest responses, most significant responses and best SNRs were in general agreement with the orientation of the source generators of the responses. Significant correlations between recording modalities were only of moderate size and only seen for few channels, suggesting that the cEEGrid records complementary but not identical information. The study also showed that an electrode behind the ear, and in very close proximity to the post-auricular muscle (E4) is susceptible to muscle artifacts such as the PAMR, whereas all other channels seemed to be largely unaffected. The cEEGrid channels also captured the well-documented finding that older participants show greater noise-floor levels. The noise-floor data also suggested that some bipolar channel configurations are systematically more prone to noise than others. On an individual level, there is a very large variability between and within participants regarding the amplitude and optimal channel configurations for the different metrics. This shows that the cEEGrid is able to capture individual differences, but also that the optimal channel orientation is not very stable across conditions and participants. Even though most participants showed reduced signal strength with the cEEGrid, there were a few participants who had similar, or stronger responses for some cEEGrid channels compared to the cap-EEG reference. The cEEGrid yielded ABR and EFR data above the statistical noise floor for most yNH and some oHI participants in their best channel configuration, which makes them generally usable for clinical, and research purposes. Nevertheless, further improvement in amplifier technology is needed to include the cEEGrid technology in a truly portable solution for AEP recordings from subcortical generators. Further research can also explore whether the availability of different channel orientations and the information captured with the cEEGrid might be of clinical interest.

## Data Availability

The datasets generated for this study are available on request to the corresponding author.

## Ethics Statement

This study was carried out in accordance with the recommendations of the local ethics committee (‘Kommission für Forschungsfolgenabschätzung und Ethik,’ University of Oldenburg, Oldenburg, Germany) with written informed consent from all subjects. All subjects gave written informed consent in accordance with the Declaration of Helsinki. The protocol was approved by the ‘Kommission für Forschungsfolgenabschätzung und Ethik,’ University of Oldenburg, Oldenburg, Germany.

## Author Contributions

MG, SD, and SV designed the experiment and analysed the protocols. MG performed data acquisition. MG analysed the data and MG and SV wrote the manuscript.

## Conflict of Interest Statement

SD has developed the cEEGrid in collaboration with TMSI, Oldenzaal, Netherlands. TMSI is the distributor of the cEEGrid. SD has no financial conflicts of interest to disclose.

The remaining authors declare that the research was conducted in the absence of any commercial or financial relationships that could be construed as a potential conflict of interest.

## Footnotes

1 www.python.org

2 https://github.com/bburan/abr
